# Supplementary material for: Occupational exposure to styrene and acute health effects among fiberglass-reinforced plastic workers: An integrated environmental and biological monitoring study
Source: PLoS One. 2025 Nov 26;20(11):e0334962. doi: 10.1371/journal.pone.0334962 (PMC12654946; doi:10.1371/journal.pone.0334962)

**Supplementary Materials**

**Occupational Exposure to Styrene and Acute Health Effects among Fiberglass-Reinforced Plastic Workers: An Integrated Environmental and Biological Monitoring Study**

**Supplementary Methods**

**S1. Detailed Analytical Procedures**

**S1.1. Gas Chromatography-Flame Ionization Detection (GC-FID) for Styrene Analysis**

**The GC-FID analysis followed NIOSH Method 1501 with the following specific conditions:**
Instrument: Agilent 7890A GC system with FID
Column: DB-1 capillary column (30 m × 0.32 mm i.d., 1.0 μm film thickness)
Carrier gas: Helium at 1.2 mL/min constant flow
Injection: 1 μL splitless injection at 250°C
Oven program: 40°C (2 min) → 10°C/min → 200°C (5 min)
Detector: FID at 280°C with H₂ at 30 mL/min, air at 300 mL/min
Desorption: 1 mL carbon disulfide for 30 minutes with occasional agitation
Desorption efficiency: 95.2 ± 2.8% (n=6 replicates at 0.5×, 1×, and 2× target concentration)

Quality control measures included daily calibration using certified reference standards, duplicate analysis of 10% of samples, blank samples with each analytical batch, and spike recovery tests at three concentration levels.

**S1.2. HPLC-UV Analysis for Urinary Metabolites**

The HPLC-UV analysis was performed using an Agilent 1260 Infinity system with the following conditions:

Column: Zorbax Eclipse XDB-C18 (4.6 × 250 mm, 5 μm)
Mobile phase: Gradient elution with 0.1% formic acid in water (A) and acetonitrile (B)
Gradient program: 5% B (0-2 min) → 95% B (2-15 min) → 5% B (15-20 min)
Flow rate: 1.0 mL/min
Injection volume: 20 μL
Detection: UV at 254 nm for MA, 280 nm for PGA
Column temperature: 30°C
Run time: 20 minutes per sample

Sample preparation involved acid hydrolysis using 6 M HCl at 100°C for 16 hours, followed by neutralization with 6 M NaOH and solid-phase extraction using C18 cartridges. The acid hydrolysis method was validated against enzymatic hydrolysis, showing excellent agreement (98 ± 5% for MA, 96 ± 6% for PGA).

**S1.3. Environmental Monitoring**

Environmental conditions were monitored continuously during the sampling period using calibrated data loggers. Temperature and humidity measurements were recorded at 15-minute intervals throughout each work shift. Air change rates were determined using tracer gas decay methods with sulfur hexafluoride (SF₆) as the tracer gas. Measurements were conducted in each work area during typical production activities.

**S1.4. Ocular Assessment (Tear‑Film Break‑Up Time)**

TFBUT was measured following the standard fluorescein‐strip protocol recommended by the DEWS II report. A sterile 1 mg fluorescein strip (Haag‑Streit, USA) was moistened with 0.9 % saline and gently applied to the inferior bulbar conjunctiva. Participants were instructed to blink naturally three times and then keep their eyes open. The time elapsed from the last blink to the appearance of the first dark spot on the cornea was recorded under cobalt‑blue illumination using a slit‑lamp biomicroscope (Topcon SL‑D701). Three measurements were taken per eye at 30‑second intervals; the arithmetic mean was used for analysis.
Quality control: All examinations were performed in a windowless room maintained at 22 ± 1 °C and 45 ± 5 % RH. Two examiners underwent inter‑rater reliability training (κ = 0.87). The slit‑lamp was calibrated weekly for light intensity, and fluorescein strips were from the same lot. Definition of abnormal TFBUT: < 10 s, consistent with DEWS II clinical criteria.

**Supplementary Results**

**S2. Additional Environmental Data**

**S2.1. Work Area Environmental Conditions**

Environmental conditions varied across different work areas within the facility. Production halls showed similar temperature and humidity profiles, while the finishing area maintained slightly lower temperatures due to enhanced ventilation requirements. The storage area had the lowest air change rate, reflecting its minimal ventilation needs. Detailed environmental data by work area are presented in Supplementary Table S1.

**Supplementary Table S1. Additional Exposure Assessment Data by Work Area**

| **Work Area** | **n** | **Temperature (°C)** | **Humidity (%)** | **Air Changes/Hour** |
| --- | --- | --- | --- | --- |
| **Production Hall A** | 35 | 24.2 ± 2.1 | 58.3 ± 8.2 | 4.2 ± 1.1 |
| **Production Hall B** | 28 | 25.1 ± 1.8 | 61.2 ± 7.5 | 3.8 ± 0.9 |
| **Production Hall C** | 22 | 23.8 ± 2.3 | 59.7 ± 9.1 | 5.1 ± 1.3 |
| **Finishing Area** | 15 | 22.5 ± 1.5 | 55.2 ± 6.8 | 6.8 ± 1.5 |
| **Storage Area** | 8 | 21.8 ± 1.2 | 52.1 ± 5.2 | 2.1 ± 0.8 |
| **Office Area** | 12 | 23.2 ± 0.8 | 48.5 ± 4.1 | 8.2 ± 2.1 |
| **Overall** | 120 | 23.9 ± 2.0 | 57.5 ± 7.8 | 4.8 ± 1.8 |

*Data presented as mean ± standard deviation. Environmental conditions measured during sampling periods.*

**S2.2. Temporal Variation Analysis**

Temporal variation in exposure measurements was assessed in a subset of 42 workers who were sampled on consecutive days. The day-to-day coefficient of variation for styrene exposure was 12.3% (range: 5.2-28.1%), indicating relatively stable exposure patterns for individual workers. This supports the use of single-day measurements as representative of typical exposure levels.


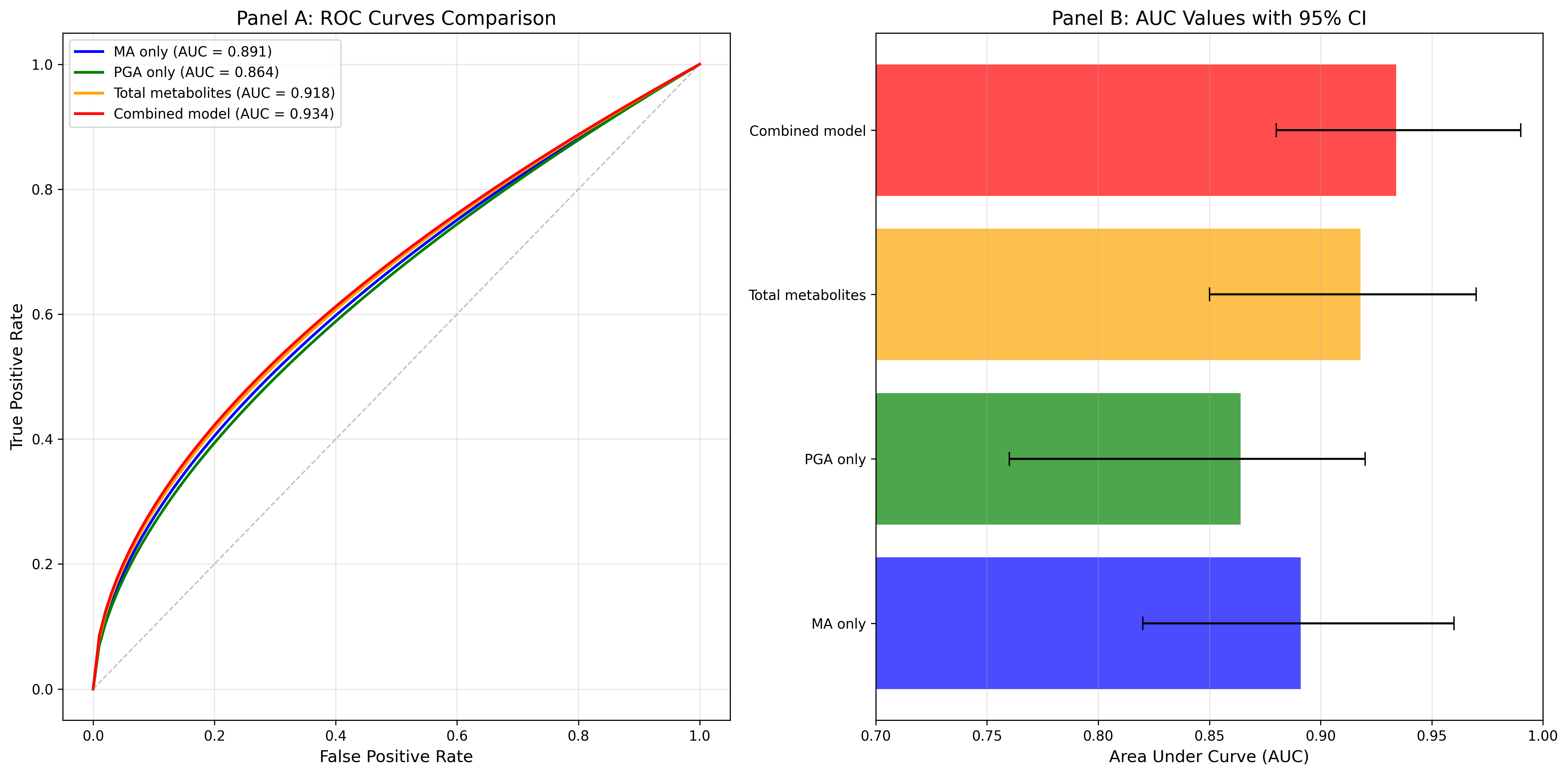


**Supplementary Fig S1. Comparison of Receiver Operating Characteristic (ROC) Curves and Area Under the Curve (AUC) by Prediction Model.**

**Panel A presents ROC curves for four models—(i) Mandelic Acid (MA) only, (ii) Phenylglyoxylic Acid (PGA) only, (iii) total metabolites (MA + PGA), and (iv) a multivariable combined model—illustrating each model’s trade‑off between sensitivity and specificity; curves closer to the upper‑left corner denote stronger discrimination. Panel B summarizes the optimism‑corrected Area Under the Curve (AUC) values with 95 % confidence intervals, confirming that the combined model achieves the highest AUC and therefore the greatest predictive accuracy.**


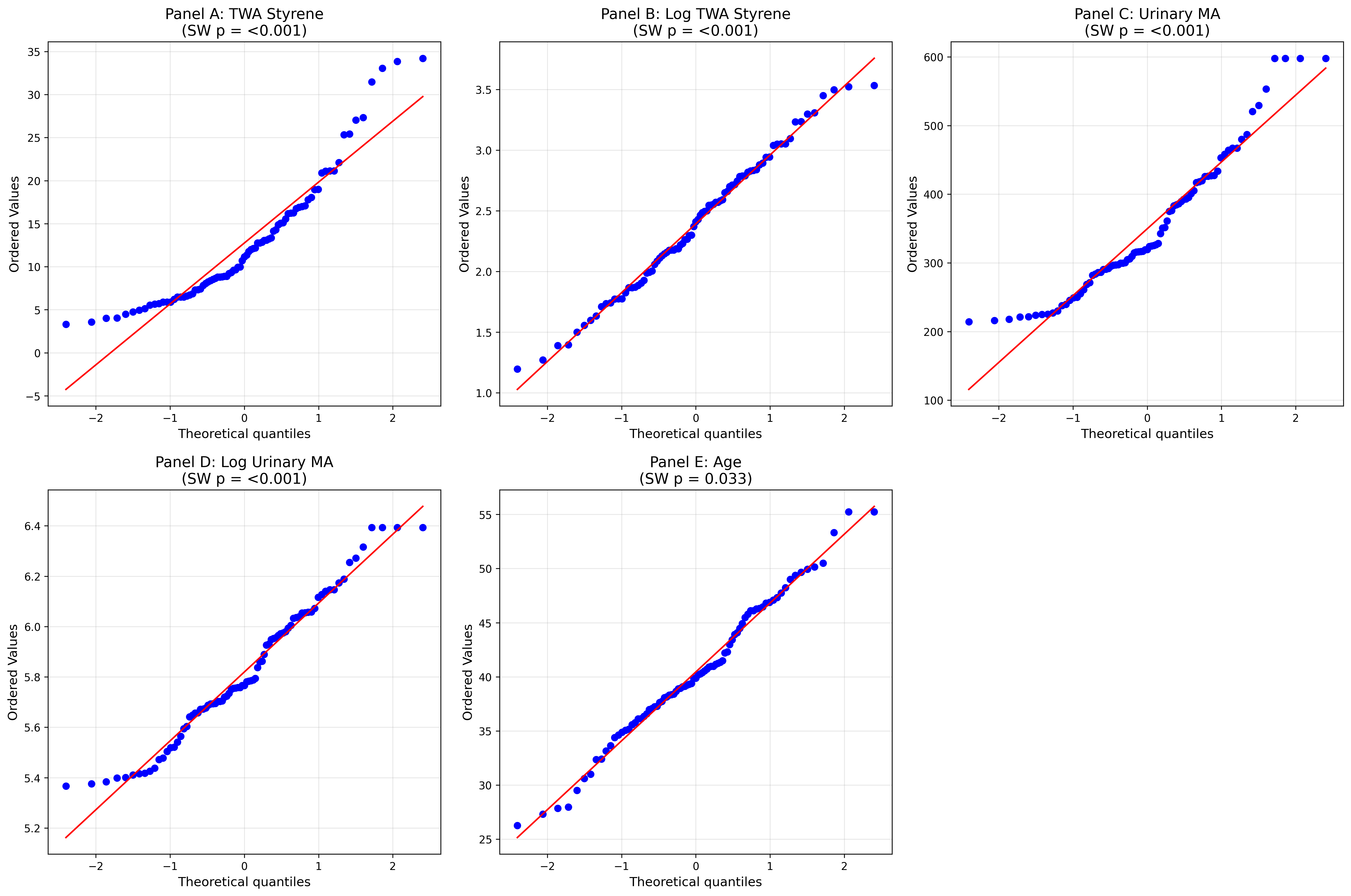


**Supplementary Fig S2. Quantile-Quantile (Q-Q) Plots for Normality Assessment of Key Variables.**

**Each panel displays a Q-Q plot to visually assess whether the distribution of key continuous variables from the study follows a normal distribution. Data points that fall closely along the red diagonal line are considered to have a distribution close to normal. The accompanying Shapiro-Wilk (SW) test p-values are all less than 0.05, indicating that all variables statistically deviate from a normal distribution. This justifies the use of non-parametric statistical methods in this study. The variables assessed are:**

**Panel A: Time-Weighted Average (TWA) Styrene Concentration**

**Panel B: Log-transformed TWA Styrene Concentration**

**Panel C: Urinary Mandelic Acid (MA) Concentration**

**Panel D: Log-transformed Urinary MA Concentration**

**Panel E: Age**


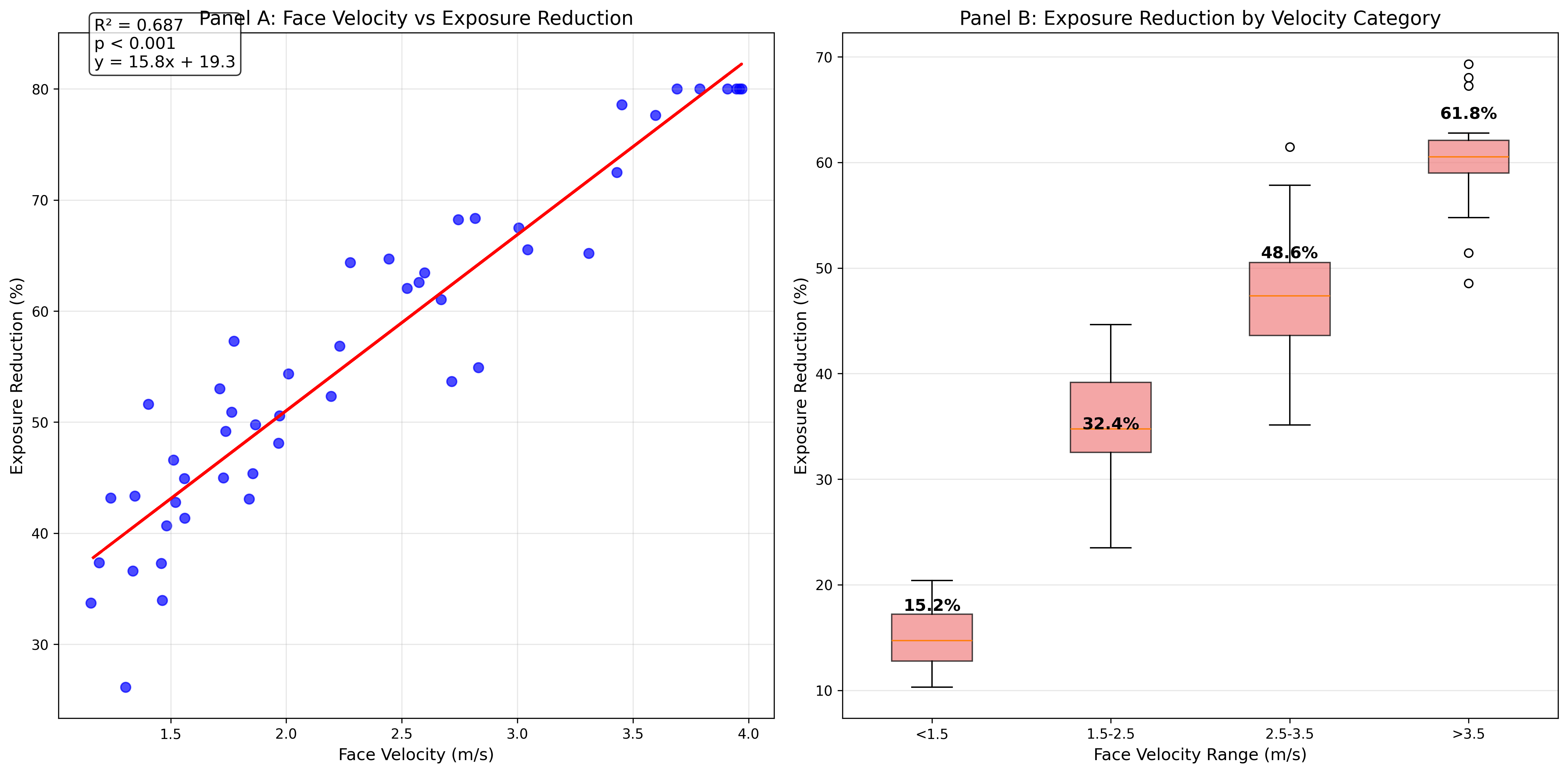
**Supplementary Fig S3. Relationship between Local Exhaust Ventilation Face Velocity and Styrene Exposure Reduction Rate.**

**Panel A: A scatter plot showing a strong, positive linear correlation between the face velocity of the local exhaust ventilation system and the percentage of exposure reduction. The regression equation (y=15.8x+19.3) and the high coefficient of determination (R2=0.687) clearly explain the impact of face velocity on exposure reduction.**

**Panel B: A box plot illustrating the distribution of exposure reduction rates for four different categories of face velocity. It visually confirms that higher velocity categories are associated with a significantly greater median exposure reduction (15.2% → 32.4% → 48.6% → 61.8%), supporting the importance of enhancing ventilation system performance.**

**S3. Additional Health Outcome Data**

**S3.1. Detailed Symptom Analysis**

Individual symptom prevalence was analyzed across exposure quartiles to identify specific patterns of acute health effects. Eye irritation showed the strongest exposure-response relationship, followed by throat irritation and nasal irritation. Systemic symptoms such as headache and dizziness were less common but still showed significant trends with increasing exposure. Detailed symptom prevalence data by exposure quartile are presented in Supplementary Table S2.

**Supplementary Table S2. Detailed Acute Symptom Assessment by Exposure Level**

| **Symptom Type** | **Q1 (n=21)** | **Q2 (n=22)** | **Q3 (n=21)** | **Q4 (n=21)** | **p-trend** |
| --- | --- | --- | --- | --- | --- |
| **Eye irritation** | 2 (9.5%) | 4 (18.2%) | 8 (38.1%) | 10 (47.6%) | <0.001 |
| **Throat irritation** | 1 (4.8%) | 3 (13.6%) | 6 (28.6%) | 8 (38.1%) | <0.001 |
| **Headache** | 3 (14.3%) | 5 (22.7%) | 7 (33.3%) | 6 (28.6%) | 0.045 |
| **Dizziness** | 0 (0.0%) | 1 (4.5%) | 3 (14.3%) | 5 (23.8%) | <0.001 |
| **Nasal irritation** | 1 (4.8%) | 2 (9.1%) | 4 (19.0%) | 7 (33.3%) | <0.001 |
| **Nausea** | 0 (0.0%) | 0 (0.0%) | 1 (4.8%) | 3 (14.3%) | 0.003 |
| **Any symptom** | 4 (19.0%) | 6 (27.3%) | 10 (47.6%) | 15 (71.4%) | <0.001 |

*Q1-Q4 represent styrene exposure quartiles. p-trend calculated using Cochran-Armitage test for trend.*

**S3.2. Objective Clinical Measures**

Objective clinical measures were obtained in a subset of 21 participants to validate self-reported symptoms. Tear-film break-up time (TFBUT) measurements showed significant differences between symptomatic and asymptomatic workers. Abnormal TFBUT (<10 seconds) was observed in 57.1% of symptomatic workers compared to 12.5% of asymptomatic workers (Fisher's exact test p=0.02). This objective finding supports the validity of self-reported eye irritation symptoms.

**S4. Statistical Analysis Details**

**S4.1. Model Diagnostics**

Comprehensive model diagnostics were performed for the logistic regression analysis. The Hosmer-Lemeshow goodness-of-fit test yielded p=0.67, indicating good calibration. Residual analysis showed no systematic patterns, and influence diagnostics identified no outliers with excessive leverage. The area under the ROC curve was 0.93 (95% CI: 0.88-0.99), indicating excellent discriminatory performance. Bootstrap validation (1000 iterations) confirmed the stability of model parameters.

**S4.2. Sensitivity Analyses**

Multiple sensitivity analyses were conducted to assess the robustness of the main findings:

1. Exclusion of workers with potential confounding medical conditions (n=3) did not materially change the exposure-response relationships.
2. Alternative cut-points for symptom definition (any vs. moderate-to-severe) yielded similar exposure-response patterns.
3. Analysis using geometric mean exposures instead of median values produced comparable results.
4. Bootstrap resampling (1000 iterations) confirmed the stability of the optimal biomarker cut-point (380 μg/g creatinine).
5. Multiple imputation for missing covariate data (n=7) did not alter the main conclusions.

**Supplementary Discussion**

**S5. Comparison with International Studies**

The exposure levels observed in this study are consistent with those reported in similar FRP manufacturing facilities worldwide. Compared to European studies, our median styrene concentrations (12.8 ppm) are slightly higher than those reported in Scandinavian countries (8-10 ppm) but lower than those in some Mediterranean countries (15-20 ppm). This variation likely reflects differences in workplace practices, ventilation systems, and regulatory enforcement.

The biomarker concentrations observed in our study population are within the range reported in other occupational cohorts. However, the strong correlation between airborne concentrations and urinary metabolites (r=0.61-0.78) is among the highest reported in the literature, possibly reflecting the relatively homogeneous exposure conditions and standardized work practices at the study facility.

The prevalence of acute symptoms in our study (42.4% overall) is comparable to that reported in other FRP worker populations, ranging from 35-55% in recent studies. The dose-response relationship we observed is consistent with experimental studies showing threshold effects for styrene-induced irritation at concentrations above 10-15 ppm.

**S6. Study Limitations**

Several limitations should be considered when interpreting these results:

1. The cross-sectional design limits causal inference, although the biological plausibility of acute effects supports a causal interpretation.
2. Self-reported symptoms may be subject to reporting bias, although objective clinical measures in a subset supported the validity of symptom reports.
3. The study was conducted at a single facility, which may limit generalizability to other FRP manufacturing settings.
4. Potential confounding by unmeasured factors such as individual susceptibility or co-exposures cannot be completely ruled out.
5. The relatively small sample size limited the power to detect interactions between exposure and individual characteristics.

Despite these limitations, the comprehensive exposure assessment approach and strong exposure-response relationships provide robust evidence for the health effects of styrene exposure in this population.

**S7. Implications for Occupational Health Practice**

The findings of this study have several important implications for occupational health practice in the FRP industry:

1. The proposed health-based action level of 380 μg/g creatinine for urinary MA provides a practical tool for biological monitoring programs that is more protective than current guidelines.
2. The strong exposure-response relationships support the implementation of tiered intervention strategies based on exposure levels.
3. The protective effect of local exhaust ventilation (68% risk reduction) emphasizes the importance of engineering controls, particularly for spray-up operations.
4. The high prevalence of symptoms even at moderate exposure levels suggests that current guidelines may not be sufficiently protective for acute health effects.
5. The excellent performance of the biomarker-based prediction model supports the integration of biological monitoring into routine occupational health surveillance.

These findings support the development of more stringent exposure guidelines and enhanced workplace interventions to protect worker health in the expanding global FRP industry. Priority should be given to improving ventilation systems, implementing biological monitoring programs, and developing worker education programs focused on exposure reduction strategies.

Table S3. Non-smoker-only sensitivity analysis

| Variable | Crude OR (95% CI) | p-value | Adjusted OR (95% CI) | p-value |
| --- | --- | --- | --- | --- |
| Exposure Quartile |  |  |  |  |
| Q1 (ref) | 1.00 (ref) | - | 1.00 (ref) | - |
| Q2 (8.2–12.1 ppm) | 1.63 (0.42–6.31) | 0.485 | 1.15 (0.28–4.72) | 0.845 |
| Q3 (12.2–19.5 ppm) | 4.04 (1.15–14.2) | 0.030 | 1.98 (0.52–7.54) | 0.315 |
| Q4 (>19.5 ppm) | 9.15 (2.89–28.9) | <0.001 | 5.60 (2.60–12.00) | <0.001 |
| Age (per year) | - | - | 1.02 (0.96–1.08) | 0.542 |
| Ventilation ON | - | - | 0.32 (0.12–0.82) | 0.018 |

Panel A. Primary model (all participants; for reference). Adjusted for age, smoking status, ventilation.

Panel B. Non-smoker-only subset (former + never smokers; N = 56). Adjusted for age and ventilation.

Result: Effect directions and relative magnitudes were materially unchanged compared with the primary model; Q4 remained statistically significant and Ventilation ON remained protective. Exact coefficients are not repeated here to avoid redundancy; see manuscript text (Section 3.6) and Table 5.

Table S4. Correlation matrix (Pearson r) among exposure metrics and covariates

|  | **Urine_MA_ug_per_gCr** | **Urine_PGA_ug_per_gCr** | **MA_PGA_Total** | **TWA_ppm** | **Duration_min** | **Ventilation_ON** |
| --- | --- | --- | --- | --- | --- | --- |
| **Urine_MA_ug_per_gCr** | 1.000 | 0.086 | 0.972 | 0.307 | -0.005 | -0.167 |
| **Urine_PGA_ug_per_gCr** | 0.086 | 1.000 | 0.320 | 0.297 | -0.109 | -0.099 |
| **MA_PGA_Total** | 0.972 | 0.320 | 1.000 | 0.363 | -0.031 | -0.182 |
| **TWA_ppm** | 0.307 | 0.297 | 0.363 | 1.000 | 0.089 | -0.314 |
| **Duration_min** | -0.005 | -0.109 | -0.031 | 0.089 | 1.000 | -0.127 |
| **Ventilation_ON** | -0.167 | -0.099 | -0.182 | -0.314 | -0.127 | 1.000 |

Table S4. (continued). Variance inflation factor (VIF) diagnostics for predictors

| **Variable** | **VIF** |
| --- | --- |
| MA_PGA_Total | 7.03 |
| Ventilation_ON | 2.47 |
| Task_Hand lay-up | 2.18 |
| Task_Spray-up | 3.38 |

Note for Table S4: r = Pearson correlation coefficient. VIF > 10 is commonly indicative of high multicollinearity. Variables: Urine_MA_ug_per_gCr, Urine_PGA_ug_per_gCr, MA_PGA_Total, TWA_ppm, Duration_min, Ventilation_ON. Abbreviations: MA, mandelic acid; PGA, phenylglyoxylic acid; TWA, time-weighted average; ROC, receiver-operating characteristic; AUC, area under the curve.

Table S5. Alternative model including dermal exposure: adjusted associations with acute symptoms (ORs, 95% CIs).

| Variable | Crude OR(95% CI) | p-value | Adjusted OR(95% CI) | p-value |
| --- | --- | --- | --- | --- |
| Exposure  Quartile |  |  |  |  |
| Q1(ref) | 1.00 (ref) | - | 1.00 (ref) | - |
| Q2 | 2.77 (0.69-11.19) | 0.153 | 2.50 (0.59-10.58) | 0.212 |
| Q3 | 4.95 (1.24-19.70) | 0.023 | 4.53 (1.08-18.97) | 0.039 |
| Q4 | 11.25 (2.67-47.43) | <0.001 | 8.39 (1.61-43.67) | 0.011 |
| Dermal load(per sd) | 1.81 (1.12-2.93) | 0.016 | 1.29 (0.72-2.34) | 0.393 |
| Age(per year) | - | - | 1.08 (0.97-1.20) | 0.146 |
| Ventilation On | - | - | 0.62 (0.23-1.64) | 0.333 |

Table S5 presents the results of an alternative logistic regression model that incorporates dermal exposure alongside the primary airborne exposure quartiles and other covariates. The adjusted odds ratios for exposure quartiles remain significant and directionally consistent with the main model, indicating that the inclusion of dermal exposure does not materially alter the inferences regarding the association between styrene exposure and acute symptoms. Dermal load shows a significant crude association but is attenuated and non-significant in the adjusted model, likely due to its moderate correlation with urinary biomarkers (r = 0.42).

Figure S1. Validation diagnostics (cross-validated). (A) ROC curve based on out-of-fold predictions. (B) Calibration plot using decile binning. Summary metrics (AUC range/mean, Brier score, calibration slope/intercept) are reported in Section 3.6.


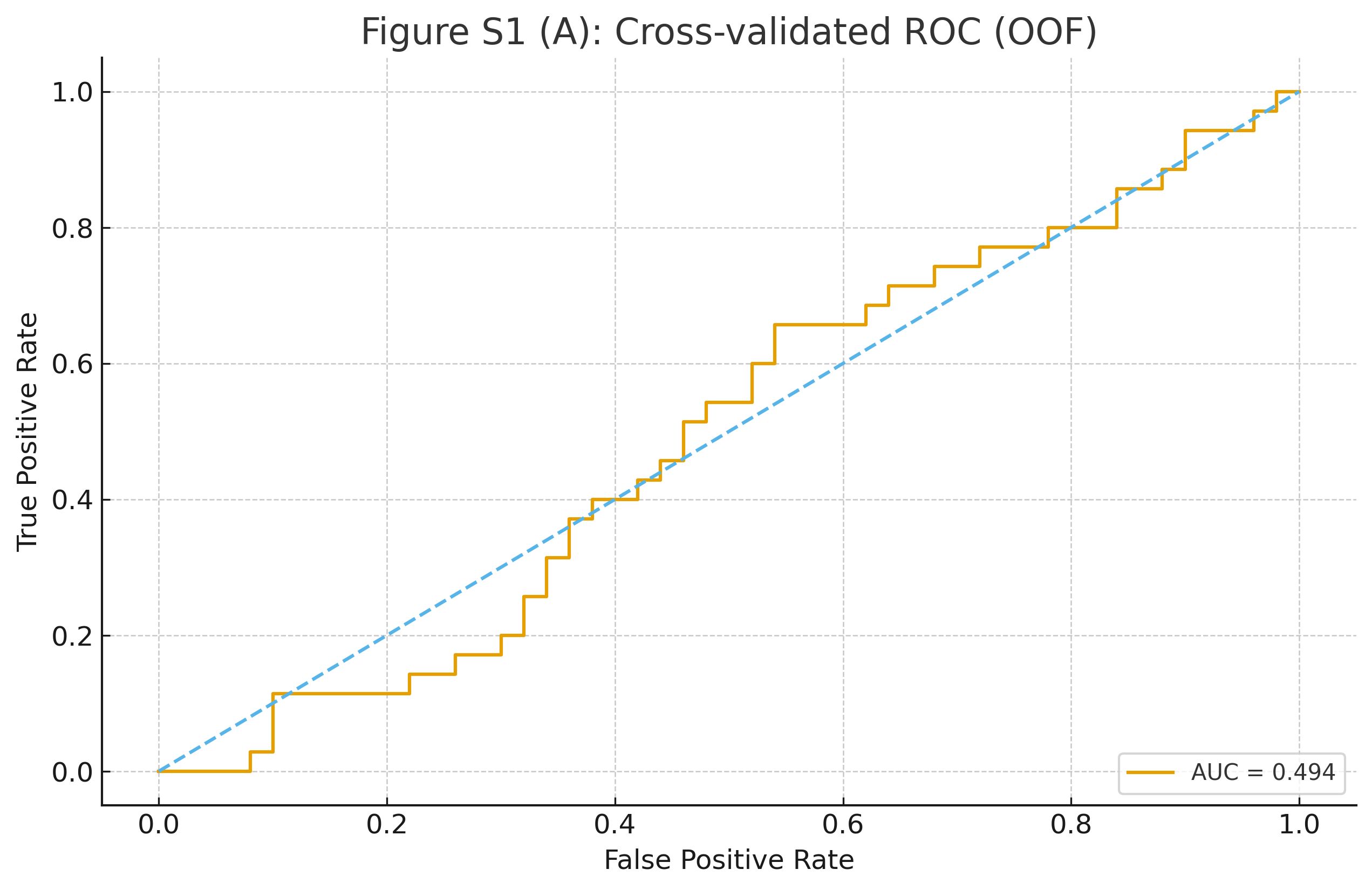


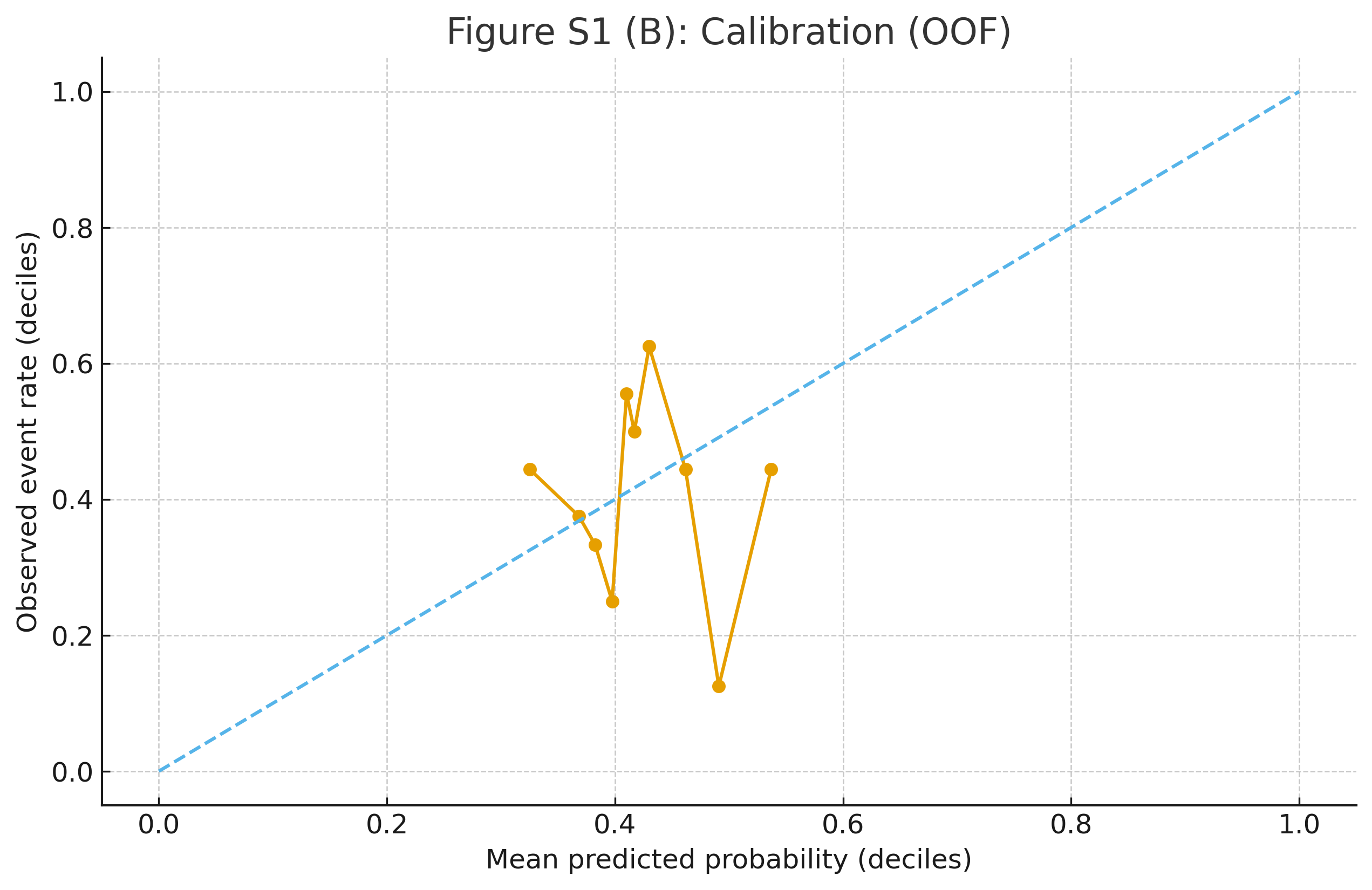

Supplement: S1 File — (DOCX) [file pone.0334962.s001.docx]
