# Supplementary material for: Occupational exposure to styrene and acute health effects among fiberglass-reinforced plastic workers: An integrated environmental and biological monitoring study
Source: PLoS One. 2025 Nov 26;20(11):e0334962. doi: 10.1371/journal.pone.0334962 (PMC12654946; doi:10.1371/journal.pone.0334962)
Supplement: Attachment — Submitted filename: 0. Response to Reviewers.docx [file pone.0334962.s003.docx]

Response to Reviewers

**Manuscript ID:** PONE-D-25-46598

**Title:** Occupational Exposure to Styrene and Acute Health Effects among Fiberglass-Reinforced Plastic Workers: An Integrated Environmental and Biological Monitoring Study

**Journal:** PLOS ONE

**Academic Editor:** Dr. Jin Su Kim

**Revision Deadline:** Nov 14, 2025, 11:59 PM

# Cover Summary (Summary of Revisions)

We thank the Academic Editor and both reviewers for their thoughtful and constructive feedback. We revised the manuscript accordingly. In brief, we:

• Adjusted language throughout to avoid causal implications, using “associated with/linked to” where appropriate for a cross-sectional design.

• Elevated the absence of detailed respirator/PPE data to a key limitation and discussed its likely bias direction (toward the null).

• Clarified smoking ascertainment and model coding; added a non-smoker–only sensitivity analysis (Table S3).

• Explained the modeling rationale regarding dermal exposure and added correlation/VIF diagnostics (Table S4) plus an alternative model including dermal exposure (Table S5).

• Reframed 0.38 mg/g creatinine (MA) as a candidate operational (“early-warning”) threshold and contrasted it with the ACGIH BEI (400 mg/g creatinine, end-of-shift); emphasized the need for external validation.

• Aligned formatting with PLOS ONE style; unified the Competing Interests statement; updated the Data Availability Statement towards open repository (DOI); verified figures via PACE.

• We also introduced a new Results subsection, 3.6 Sensitivity and Validation, summarizing spline tests, discrimination (AUC), and calibration (intercept/slope), with references to Tables S2–S5 and Fig S1.

# Responses to Journal Requirements

In the Results, we added subsection 3.6 Sensitivity and Validation to concisely report robustness checks and validation metrics (AUC, Brier, calibration), cross-referencing Tables S2–S5 and Fig S1.

**Formatting & file naming —** Aligned manuscript with PLOS ONE style samples (title/authors/affiliations pages, headings, tables/figures, file naming conventions).

**Competing Interests —** Standardized to: “The authors have declared that no competing interests exist.” and ensured consistency with the online submission form.

**Data Availability —** We thank the editorial office for the guidance on clarifying our data sharing plan. In accordance with your request, we have revised the Data Availability Statement to be more direct and to fully comply with PLOS ONE's open data policy. The updated statement now explicitly indicates that all relevant data underlying the findings are available from the Zenodo repository and provides the specific DOI for access.

**Figures (PACE) —** All figures were preflight-checked with PACE and adjusted as needed (resolution, labeling, format).

**Protocols.io (optional) —** Methods are fully detailed in the manuscript; no separate protocol DOI is needed for this submission.

# Reviewer #1 — Point-by-Point Responses

Original overall assessment: “The study is strong because it combines air monitoring, urine biomarkers, and dermal exposure… Points to improve:”

**Use association wording (cross-sectional study):**

_Reviewer comment:_ The study is cross-sectional, so you can only say styrene exposure is “associated with” symptoms, not that it directly “causes” them.

_Response:_ We agree. We systematically revised wording to avoid causal language. We now use “associated with/linked to” in the Abstract conclusion and throughout the Discussion (Limitations).

Changes in manuscript:

• Abstract (last sentence) revised; Discussion (Limitations) revised.

**PPE information as limitation:**

_Reviewer comment:_ The study does not include information on the use of personal protective equipment (like respirators)… Please highlight this more clearly as a limitation.

_Response:_ We concur. We elevated the absence of detailed respirator/PPE data to a key limitation and discussed likely bias direction (tending toward the null). Where feasible, we used task/ventilation proxies; inferences were unchanged (Table S2).

Changes in manuscript:

• Discussion limitation paragraph added; Table S2 added (proxy sensitivity).

**Smoking measurement details:**

_Reviewer comment:_ Smoking was considered, but more detail on how it was measured would improve the paper.

_Response:_ We clarified how smoking was ascertained and coded (e.g., current/former/never; or current vs. non-current). We added a non-smoker–only sensitivity analysis with consistent results (Table S3).

Changes in manuscript:

• Methods (Covariates/Statistical Analysis) updated; Table S3 added (non-smokers).

**Generalizability (single factory; male workers):**

_Reviewer comment:_ The study was done in one factory and only on male workers, so the findings may not apply to all workers. This should be explained in the discussion.

_Response:_ We expanded the Discussion to explicitly acknowledge limited external validity and recommend replication across multi-site, mixed-sex cohorts with differing process controls.

Changes in manuscript:

• Discussion paragraph on external validity expanded.

**Dermal exposure modeling clarity:**

_Reviewer comment:_ Dermal exposure was measured but not fully included in the final model. Please explain this more clearly.

_Response:_ We explained our modeling rationale (airborne/dermal correlation; potential pathway to urinary metabolites) to avoid multicollinearity and overadjustment. We added correlation/VIF diagnostics (Table S4) and an alternative model including dermal exposure (Table S5), with materially unchanged inferences.

Changes in manuscript:

• Methods modeling rationale added; Tables S4–S5 added.

**0.38 mg/g threshold vs ACGIH BEI and practical meaning:**

_Reviewer comment:_ The proposed new biomarker action level (0.38 mg/g creatinine) is interesting, but explain how it compares to the current ACGIH limit and what it would mean in practice.

_Response:_ We reframed 0.38 mg/g creatinine (MA) as a candidate operational threshold for early flagging and contrasted it with the ACGIH BEI (400 mg/g creatinine, end-of-shift), noting that BEIs are exposure indices rather than health-effect thresholds. We clarified practical implications and emphasized the need for external validation.

Changes in manuscript:

• Discussion implications revised; Abstract implications sentence adjusted.

# Reviewer #2 — Point-by-Point Response

_Reviewer comment:_ Single point measurement on a narrow worker range; repeated measures are needed because prior exposures may affect same-day absorption.

_Response:_ We agree and expanded the Discussion accordingly. Urinary MA/PGA predominantly reflect recent exposure (hour-scale effective half-times), but multi-compartment elimination means carryover from prior exposures cannot be excluded. This underscores the value of repeated biomonitoring in longitudinal designs. Where feasible, we adjusted for shift timing/previous shift in a sensitivity check; inferences remained unchanged.

Changes in manuscript:

• Discussion paragraph on elimination kinetics and need for repeated measures added.

• Methods note on sensitivity adjustment for previous shift/shift timing added.

# New/Updated Materials Included with the Revision

• Revised Manuscript with Track Changes (Manuscript_tracked.docx).

• Clean Manuscript (Manuscript_clean.docx).

• Response to Reviewers (this document).

• Supplementary: Table S2 (proxy/PPE), Table S3 (non-smokers), Table S4 (correlations/VIFs), Table S5 (dermal-included model), Fig S1 (optional calibration/ROC).

• PACE-verified figures.

• Data & Code package (repository DOI to be inserted in DAS).
